# *Salmonella enterica* serovar Cerro displays a phylogenetic structure and genomic features consistent with virulence attenuation and adaptation to cattle

**DOI:** 10.3389/fmicb.2022.1005215

**Published:** 2022-11-30

**Authors:** Alexa R. Cohn, Renato H. Orsi, Laura M. Carroll, Jingqiu Liao, Martin Wiedmann, Rachel A. Cheng

**Affiliations:** ^1^Department of Food Science, Cornell University, Ithaca, NY, United States; ^2^Structural and Computational Biology Unit, European Molecular Biology Laboratory, Heidelberg, Germany; ^3^Department of Civil and Environmental Engineering, Virginia Tech, Blacksburg, VA, United States

**Keywords:** *Salmonella*, food safety, evolution, genomics, pathogen

## Abstract

*Salmonella enterica* subsp*. enterica* (*S.*) serovar Cerro is rarely isolated from human clinical cases of salmonellosis but represents the most common serovar isolated from cattle without clinical signs of illness in the United States. In this study, using a large, diverse set of 316 isolates, we utilized genomic methods to further elucidate the evolutionary history of *S.* Cerro and to identify genomic features associated with its apparent virulence attenuation in humans. Phylogenetic analyses showed that within this polyphyletic serovar, 98.4% of isolates (311/316) represent a monophyletic clade within section Typhi and the remaining 1.6% of isolates (5/316) form a monophyletic clade within subspecies *enterica* Clade A1. Of the section Typhi *S.* Cerro isolates, 93.2% of isolates (290/311) clustered into a large clonal clade comprised of predominantly sequence type (ST) 367 cattle and environmental isolates, while the remaining 6.8% of isolates (21/311), primarily from human clinical sources, clustered outside of this clonal clade. A tip-dated phylogeny of *S.* Cerro ST367 identified two major clades (I and II), one of which overwhelmingly consisted of cattle isolates that share a most recent common ancestor that existed *circa* 1975. Gene presence/absence and rarefaction curve analyses suggested that the pangenome of section Typhi *S.* Cerro is open, potentially reflecting the gain/loss of prophage; human isolates contained the most open pangenome, while cattle isolates had the least open pangenome. Hypothetically disrupted coding sequences (HDCs) displayed clade-specific losses of intact *speC* and *sopA* virulence genes within the large clonal *S.* Cerro clade, while loss of intact *vgrG*, *araH*, and *vapC* occurred in all section Typhi *S.* Cerro isolates. Further phenotypic analysis suggested that the presence of a premature stop codon in *speC* does not abolish ornithine decarboxylase activity in *S.* Cerro, likely due to the activity of the second ornithine decarboxylase encoded by *speF*, which remained intact in all isolates. Overall, our study identifies specific genomic features associated with *S.* Cerro’s infrequent isolation from humans and its apparent adaptation to cattle, which has broader implications for informing our understanding of the evolutionary events facilitating host adaptation in *Salmonella*.

## Introduction

*Salmonella* is a major cause of foodborne illness in humans and is responsible for an estimated 1.35 million illnesses, 26,500 hospitalizations, and 420 deaths in the United States each year (cdc.gov, 2018b). The genus *Salmonella* is comprised of two species: *S. enterica* and *S. bongori* (Brenner et al., 2000); *S. enterica* is further divided into six recognized subspecies [*enterica, salamae, arizonae, diarizonae, houtenae,* and *indica* (Brenner et al., 2000)] and four proposed novel subspecies (VII, A, B, and C; Alikhan et al., 2018). Further, there are 2,659 recognized serovars within the genus *Salmonella* that differ in their host ranges and severity of disease that they cause (Brenner et al., 2000; Jones et al., 2008; Issenhuth-Jeanjean et al., 2014). *Salmonella enterica* subsp. *enterica* serovars (hereafter referred to as “*S.*”), which represent the most clinically relevant subspecies, can be further divided based on their disease presentation; typhoidal serovars (e.g., *S.* Typhi) cause the systemic illness typhoid fever, while nontyphoidal serovars (e.g., *S.* Typhimurium) typically cause a self-limiting gastroenteritis, but can be associated with extraintestinal disease (Jones et al., 2008). Furthermore, nontyphoidal *Salmonella* (NTS) serovars differ in their ability to cause disease in various hosts.

*S.* Cerro is a NTS serovar that has become increasingly associated with dairy and beef cattle operations in the United States, representing the second most commonly isolated serovar from cattle with clinical signs of salmonellosis and the most common serovar isolated from cattle without clinical signs of salmonellosis in the United States (cdc.gov, 2018a). Interestingly, many studies have highlighted the common isolation of *S.* Cerro from cattle without clinical signs of salmonellosis. Cummings et al. found that 20 of 57 New York State dairy herds were positive for *S.* Cerro (Cummings et al., 2010). Similarly, in a study of United States and Mexican beef cattle presented for slaughter in Texas, Nickelson et al. found that 52% of cattle lymph nodes were positive for *Salmonella*; serotyping of these samples revealed that 21.6% of the *Salmonella* isolated were *S.* Cerro (Nickelson et al., 2019). Finally, a meta-analysis of studies published between 2000 and 2017 found that *S.* Cerro is one of the top 10 most commonly isolated serovars from apparently healthy cattle globally (Gutema et al., 2019). Despite its common isolation from cattle in the United States, *S.* Cerro was only responsible for 38 culture-confirmed human illnesses in 2016 (0.08% of total culture-confirmed human illnesses), compared to 4,581 *S.* Typhimurium isolates from human clinical specimens (cdc.gov, 2018b).

Multiple studies have attempted to explain why *S.* Cerro is commonly associated with cattle but infrequently causes human illness. Rodriguez-Rivera et al. found that *S.* Cerro sequence type (ST) 367 isolates (i) contained a premature stop codon (PMSC) in the *Salmonella* pathogenicity island (SPI) 1 effector, *sopA*, (ii) were missing genes in SPIs 10, 12, and 13, and (iii) displayed a stepwise loss of D-alanine transport genes (Rodriguez-Rivera et al., 2014). Additionally, Kovac et al. found that a subclade of *S.* Cerro ST367 contained PMSCs and deletions in the thiosulfate reductase, *phsA*, resulting in loss of function (Kovac et al., 2017). Finally, using a comparative transcriptomics approach, we have previously shown that compared to isolates representing *S.* Javiana and *S.* Typhimurium, *S.* Cerro displays significantly lower transcript abundances of SPI-1 genes when grown to late exponential phase in Luria-Bertani (LB) broth (Cohn et al., 2021b). While these findings identified potential explanations for *S.* Cerro’s apparent adaptation to cattle and attenuated virulence in humans, they have focused primarily on ST367 isolates, and therefore our understanding of the genetic diversity of isolates within *S.* Cerro remains incomplete.

Additionally, degradation of metabolic pathways is a common feature of many bacterial pathogens and may indicate adaptation to a specific host or niche (Parkhill et al., 2001; Moran, 2002; Gómez-Valero et al., 2007; Diop et al., 2018). Applying this framework to *Salmonella*, a few studies have identified degradation of metabolic pathways and hypothetically disrupted coding sequences (HDCs) in host-adapted *Salmonella* serovars that cause extraintestinal disease. On average, host-adapted extraintestinal serovars (e.g., *S.* Dublin) contained more HDCs than broad host range gastrointestinal serovars (e.g., *S.* Typhimurium); specifically, type III secretion system effector proteins, fimbrial adhesins, and motility- and chemotaxis-related genes were degraded in extraintestinal serovars (Nuccio et al., 2014). Similarly, Langridge et al. found that the genomes of host-restricted *S.* Gallinarum biovars Gallinarum and Pullorum contained more HDCs than *S.* Dublin and *S.* Enteritidis, with many of these HDCs occurring within metabolic pathways including vitamin B12 biosynthesis and allantoin degradation (Langridge et al., 2015). However, degradation of metabolic pathways in serovars that are associated with a specific host but do not cause extraintestinal disease, like *S.* Cerro, have not been characterized.

In this study, we utilized comparative genomic analyses to characterize a large dataset of *S.* Cerro isolates to (i) expand our understanding of host adapted NTS serovars that do not cause extraintestinal disease in their primary host, and (ii) elucidate mechanisms involved in *S.* Cerro’s apparent virulence attenuation in humans.

## Materials and methods

### Isolate selection and whole genome sequence assembly

Metadata of all 316 *S.* Cerro isolates in the NCBI Pathogen Detection database^1^ were downloaded [accessed 1/20/20; (Sayers et al., 2021)]. A random number generator was used to select one isolate to represent each single nucleotide polymorphism (SNP) cluster; all isolates without assigned SNP clusters were also included in these analyses and are hereafter referred to as singletons. Additionally, we included 86 isolates from a previously defined strain set (Kovac et al., 2017). To allow for clade identification, we also included a previously published dataset of genomes representing of 235 *S. enterica* subsp. *enterica* serovars and the five other *S. enterica* subspecies (Gaballa et al., 2021). The full list of isolates used in these analyses can be found in Supplementary File 1. Assemblies were downloaded from NCBI through the Read Assembly and Annotation Pipeline Tool (RAPT) v. 0.2.0 (NCBI, 2020), which includes assembly with SKESA v. 2.4.0 (Souvorov et al., 2018) and annotation with the Prokaryotic Genome Annotation Pipeline (PGAP) v. 2020-03-11 (Tatusova et al., 2016). Quality of assemblies was assessed with QUAST v. 5.1.0rc1 (Gurevich et al., 2013). SISTR v. 1.0.2 (Yoshida et al., 2016) was used to confirm the serotype listed on NCBI. Sequence types of *S.* Cerro isolates were determined with mlst v. 2.16.1^2^ using a seven gene *S. enterica* typing scheme (accessed 8/27/21).

### Initial phylogenetic tree construction

**Figure 1:**
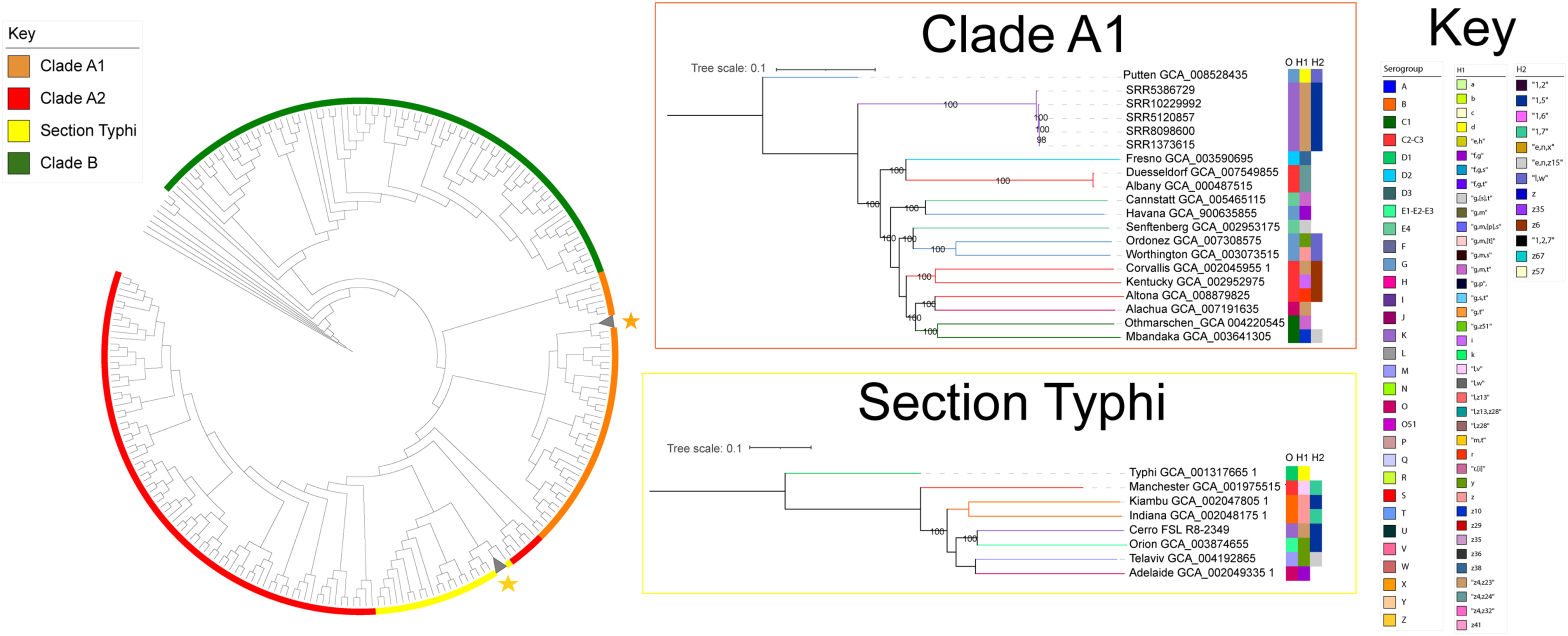
*S.* Cerro is polyphyletic and the majority of isolates cluster within the *Salmonella* section Typhi lineage. Maximum likelihood phylogenetic tree constructed from 12,238 core SNPs for representative isolates of *S.* Cerro and isolates representing 235 *S. enterica* subsp. *enterica* serovars and five *Salmonella* subspecies (Gaballa et al., 2021); branch lengths are not shown. Clade designations shown are based on those defined previously (Worley et al., 2018). *Salmonella enterica* subsp. *arizonae* was used as an outgroup to root the phylogeny. Maximum likelihood trees of the sub-clades in which *S.* Cerro isolates cluster are also shown. The Clade A1 tree was constructed from 60,135 core SNPs and the section Typhi tree was constructed from 46,278 core SNPs. Phylogenies are rooted by outgroup (the clade A1 phylogeny uses *S.* Putten GCA_008528435 as an outgroup, the section Typhi phylogeny uses *S.* Typhi GCA_001317665.1 as an outgroup; bootstrap values represent the average of 1,000 bootstrap repetitions).

kSNP v. 3.1 was used to identify core SNPs among the 316 *S.* Cerro isolates and isolates representing 235 serovars and five *S. enterica* subspecies using a *k*-mer size of 19 nt, as determined with Kchooser (Gardner and Hall, 2013). RAxML v. 8.2.12 was used to construct a maximum likelihood phylogeny of core SNPs using the GTRCAT model with Lewis ascertainment bias and 100 bootstrap replicates (Stamatakis, 2014) using *S. enterica* subsp. *arizonae* GCA_000018625.1 as an outgroup as it has been shown to be the earliest diverging *S. enterica* subspecies (Verma and Reeves, 1989; Boyd et al., 1996; Worley et al., 2018). Additional trees containing serovars that clustered with the two *S.* Cerro lineages were constructed by identifying core SNPs with kSNP v. 3.1 using a *k-*mer size of 19 nt (Gardner and Hall, 2013). RAxML v. 8.2.12 was used to construct a maximum likelihood phylogeny of core SNPs using the GTRCAT model with Lewis ascertainment bias and 1,000 bootstrap replicates (Stamatakis, 2014). As the initial phylogeny showed that 311 out of 316 *S.* Cerro isolates clustered within section Typhi and shared a most recent common ancestor (MRCA) with *S.* Orion GCA_003874655 (Figure 1), kSNP v. 3.1 (Gardner and Hall, 2013) was re-run to identify core SNPs among these 311 section Typhi *S.* Cerro isolates with *S.* Orion GCA_003874655 as an outgroup using a *k-*mer size of 19 nt. A phylogeny was inferred using the method described above for the phylogenetic tree constructed of all *S.* Cerro isolates, 235 serovars, and five *S. enterica* subspecies. Phylogenies were visualized and edited using the Interactive Tree of Life v. 5 (Letunic and Bork, 2019).

### Reference-based variant calling and tip-dated phylogeny construction

*S.* Cerro ST367 isolates were selected for reference-based variant calling and tip-dated phylogeny construction by stratified random sampling. Briefly, *S.* Cerro isolates in the NCBI Pathogen Detection database (accessed 09/01/2021) that were confirmed to be ST367 using mlst v. 2.16.1 (see footnote 2) were stratified into four date ranges (2017–2021, 2012–2016, 2007–2011, and prior to 2007), and a random number generator was used to select 25 isolates per date range (a full list of isolates can be found in Supplementary File 1). Reference-based variant calling was performed with Snippy v. 4.3.6^3^ using *S.* Cerro strain 87 (accession number GCA_001941405.1) as a reference genome (representing a ST367 closed genome) and default settings. Sites of recombination were identified in the resulting alignment with Gubbins v. 2.3.4 (Croucher et al., 2014) and snp-sites v. 2.5.1 (Page et al., 2016) was used to retrieve core SNP sites from the Snippy alignment. The resulting SNPs were used to construct a maximum likelihood phylogeny with FastTree (Price et al., 2010) for use in temporal regression analyses (Supplementary Figure S1).

Temporal signals were assessed using root-to-tip regression analysis with TempEst v. 1.5.3 (Rambaut et al., 2016) and the date randomization test (Duchêne et al., 2015). As the R-squared correlation coefficient (i.e., the relationship between time and genetic distance) determined with the root-to-tip regression analysis using a best-fitting root was deemed strong enough to perform a date randomization test to confirm the temporal signal (*R* > 0.10), we used the R package TipDatingBeast (Rieux and Khatchikian, 2017) to generate ten permutations of randomized sample dates. BEAST v. 2.5.2 (Bouckaert et al., 2019) was used to estimate evolutionary rates of alignments with randomized dates and the alignment with the true sample dates. The 95% highest posterior density (HPD) of the substitution rate of the alignment with true sample dates fell outside the 95% HPD for the randomized dates alignments, deeming that the temporal signal was sufficient (Supplementary Figure S2).

bModelTest (Bouckaert and Drummond, 2017) identified Model 23 as the best-fitting nucleotide substitution model for SNPs in the alignment. BEAST v. 2.5.2 (Bouckaert et al., 2019) was used to construct a tip-dated phylogenetic tree using the Model 23 nucleotide substitution model, relaxed log-normal molecular clock, coalescent constant size population model, and a substitution rate prior set to 4.2 × 10^−7^ substitutions/site/year, as these parameters were determined to be the best model combination in two prior *S.* Cerro ST367 studies (Rodriguez-Rivera et al., 2014; Kovac et al., 2017). An ascertainment bias correction was included to account for the use of solely variant sites as previously described (Kovac et al., 2017). Trees were constructed with chain lengths of 400 million generations and parameters were logged every 400,000 generations. This model combination was run five times with different random seeds (i.e., (i) 20,220,505, (ii) 20,220,507, (iii) 20,220,509, (iv) 20,220,511, and (v) 20,220,513). The log and tree files of individual runs were combined in LogCombiner v. 2.5.2 (Bouckaert et al., 2019), removing the first 10% of each Markov chain Monte Carlo (MCMC) run as burn-in. Output statistics and traces were analyzed in Tracer v. 1.7.2 (Rambaut et al., 2018) and the effective sample size of each test statistic was confirmed to be >200. The final tree represents a maximum clade credibility tree of the combination of the five runs and was annotated in TreeAnnotator v. 2.5.2 (Bouckaert et al., 2019) and edited in FigTree v. 1.4.4.

### Inference of the *S.* Cerro pangenome

Draft genomes of section Typhi *S.* Cerro isolates annotated with PGAP v. 2020-03-11 (Tatusova et al., 2016) were used as input for Panaroo v. 1.2.3 (Tonkin-Hill et al., 2020) for the inference of the section Typhi *S.* Cerro pangenome using the default sequence identity of 95%, default protein family threshold of 70%, and the “strict” stringency mode. Resulting pangenomes were analyzed and visualized using the R package Pagoo (Ferrés and Iraola, 2020). A gene was defined as core if it was detected in every isolate in the comparison (*n* = 311), shell genes were defined as those detected in 15–99% of isolates in the comparison, and cloud genes were defined as those detected in <15% of isolates in the comparison. The Power Law model was used to determine the openness of the *S.* Cerro pangenome. The Power Law model is described by the formula Δ*n* = *κN* – *α*, where Δ*n* represents the number of newly added genes, *N* is the number of genomes included in the study, and *κ* and *α* are fitting parameters (Tettelin et al., 2008). If *α* > 1, the pangenome is considered closed, whereas if *α* < 1, the pangenome is considered open.

To determine if the pangenome openness differed significantly in isolates from different isolation sources (i.e., human clinical, environmental, and cattle), we performed a permutation analysis by randomly selecting 15 isolates from each category 100 times per category and calculating the Power Law Model for each iteration. The resulting groups of *α* values from each Power Law Model calculation were compared with ANOVA and post-hoc Tukey Honest Significant Differences to determine significance.

Scoary v. 1.6.14 (Brynildsrud et al., 2016) was used to perform genome wide association studies based on isolation source (human clinical, cattle, or environmental) and sequence type (i.e., ST367 vs. other sequence types) using the false discovery rate (FDR) for multiple comparisons correction (Benjamini and Hochberg, 1995). Prophage regions were predicted with PHASTER (Arndt et al., 2016) in representative ST367 (GCA_001941405.1) and non-ST367 (GCA_10567865.1); these genomes were selected because they contain the fewest number of contigs (1 and 31, respectively) and had the highest N50 values (46,024,239 and 399,393, respectively). Prophage regions detected using PHASTER were then searched in all section Typhi *S.* Cerro genomes using a stand-alone database with nucleotide BLAST v. 2.9.0 (Camacho et al., 2009), using >80% identity and query coverage as cutoffs.

### Determination of hypothetically disrupted coding sequences

Genbank files of draft genomes annotated with PGAP v. 2020-03-11 (Tatusova et al., 2016) were converted to FASTA file format using the Genbank to FASTA Python script^4^ and coding sequences containing the qualifier “/pseudo” were extracted from the resulting file. Multiple sequence alignments of putative HDCs were constructed in Geneious (Auckland, New Zealand) using the Geneious aligner. Over- or underrepresentation of HDCs based on isolation source or clade was determined using Fisher’s exact tests with FDR adjustment.

### Phenotypic detection of ornithine decarboxylase activity

Stock cultures of *S.* Cerro FSL R8-2349 (contains a PMSC in *speC*), *S.* Cerro FSL M8-0630 (contains an intact version of *speC*), *S.* Typhimurium ATCC 14028S (positive control for ornithine decarboxylase activity), and *S.* Typhi FSL R6-0541 (negative control for ornithine decarboxylase activity) were streaked from Luria-Bertani (LB; 5 g NaCl/L) with 15% (v/v) glycerol stocks onto LB agar and incubated at 37°C for 24 h to obtain single colonies. Individual colonies were resuspended in 100 μl phosphate-buffered saline (PBS) and a 1 μl inoculating loop was used to inoculate Remel Motility-Indole-Ornithine (MIO) Medium (ThermoFisher, Waltham, MA) with the suspended culture. Inoculated MIO Medium was incubated at 37°C for 24 h. Isolates were considered positive for ornithine decarboxylase activity if the MIO Medium was purple and negative if the medium was yellow.

### Data availability

Accession numbers of WGS data of isolates included in this study can be found in Supplementary File 1. Computational log files and scripts are available on GitHub.^5^

## Results

### The *S.* Cerro lineage within subspecies *enterica* section Typhi represents the majority of isolates with this serotype

Previous analyses have shown that *S.* Cerro is polyphyletic (Worley et al., 2018; Cohn et al., 2021b), but have not analyzed the prevalence of isolates within these different clades. Therefore, we first sought to elucidate the population structure of *S.* Cerro. Maximum likelihood phylogenetic analysis of 12,238 core SNPs among 316 *S.* Cerro isolates, 235 representative isolates of common *Salmonella* serovars, and five *S. enterica* subspecies (Supplementary File 1) confirmed the presence of two lineages of *S.* Cerro: one clustered with *S. enterica* subsp. *enterica* clade A1 serovars and one clustered with serovars in section Typhi (Figure 1; Worley et al., 2018). The majority of *S.* Cerro isolates clustered within subsp. *enterica* section Typhi (311 of 316 *S.* Cerro isolates included in this analysis, representing 97 unique SNP clusters consisting of a total of 922 isolates) and shared a MRCA with *S.* Orion (antigenic formula I 3,{10}{15}{15,34}:y:1,5). The remaining five isolates (representing five unique SNP clusters that combined included a total of 116 isolates formed a monophyletic clade within subsp. *enterica* Clade A1, but we were unable to definitively infer a MRCA with *S.* Putten (antigenic formula I 13,23:d:l,w) based on low bootstrap support; Figure 1). Overall, although *S.* Cerro is polyphyletic, isolates within section Typhi represent the predominant clade within this serovar and we therefore focused our analyses on isolates within this lineage.

### The section Typhi *S.* Cerro lineage includes predominantly ST367 isolates from cattle, while human clinical isolates are overrepresented among more divergent lineages representing different sequence types

**Figure 2:**
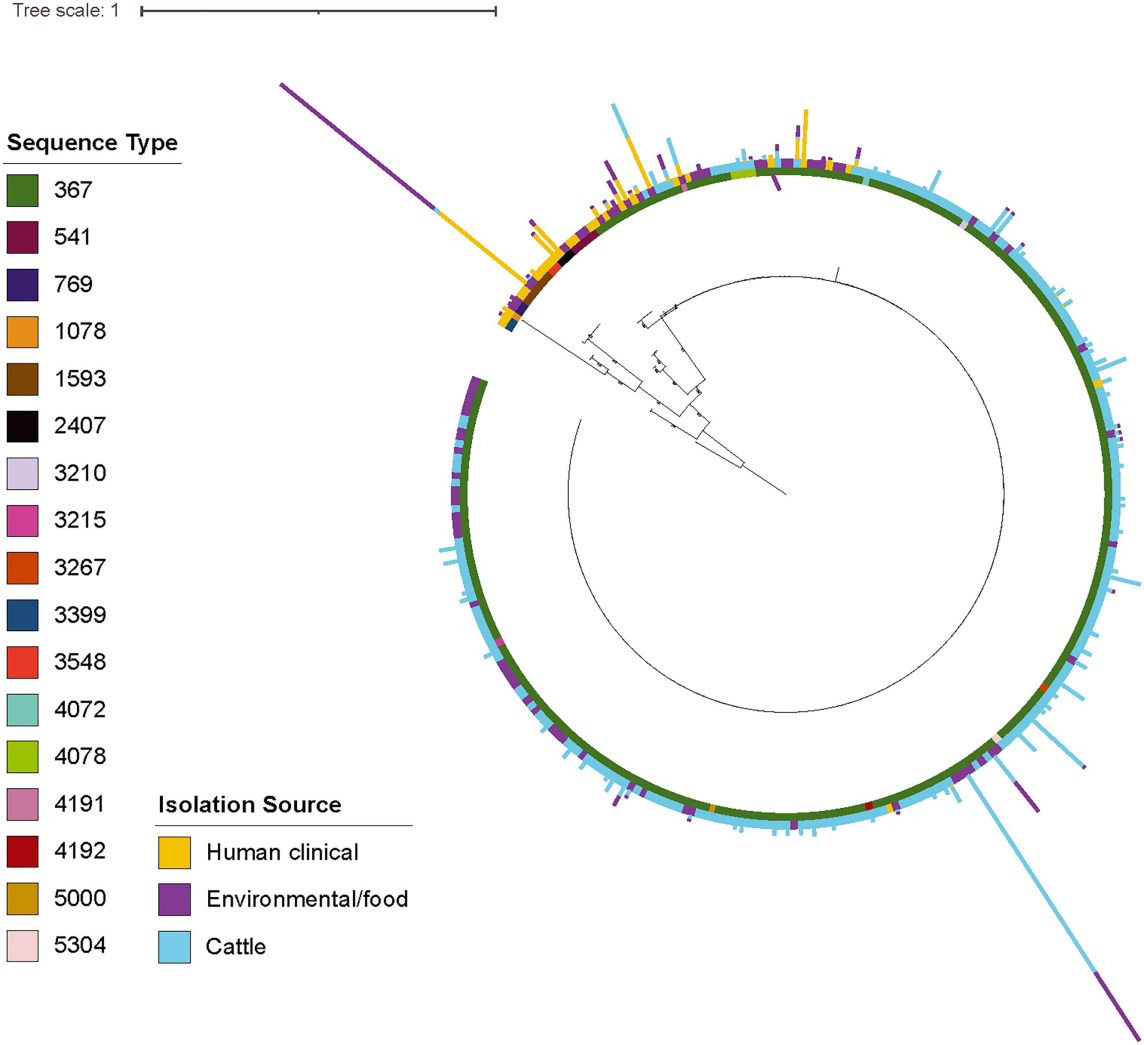
The majority of *S.* Cerro cattle isolates cluster within a large, clonal clade within subsp. *enterica* section Typhi while human clinical isolates represent more diverse lineages. Maximum likelihood phylogenetic tree constructed from core SNPs identified with kSNP3 (Gardner and Hall, 2013) for (i) representative isolates of section Typhi *S.* Cerro SNP clusters included in NCBI Pathogen Detection as of 1/20/20 and (ii) a previously published set of *S.* Cerro isolates (Kovac et al., 2017); *S.* Orion GCA_003874655 was included as an outgroup to root the phylogeny as it shares a MRCA with section Typhi *S.* Cerro. Bootstrap values represent the average of 100 bootstrap repetitions and only bootstraps >70 are shown for clades containing >5 isolates. Sequence types, as determined using the 7-gene MLST scheme, are shown in the inner color ring, isolation source (i.e., human clinical, food/environmental, and cattle) is shown in the outer ring, and the number of isolates from each isolation source (i.e., human clinical, food/environmental, and cattle) for the representative isolates’ SNP clusters are represented by the bar graphs shown external to the leaves.

To further elucidate the population structure of isolates in the section Typhi *S.* Cerro lineage, we next constructed a phylogeny of the 311 section Typhi *S.* Cerro isolates (Figure 2). Most of the isolates in this lineage clustered within a large, clonal clade (290 of 311 isolates), with the remaining 21 isolates representing more diverse lineages with longer branch lengths (Figure 2). Multi-locus sequence typing (MLST) analysis showed that 96% of *S.* Cerro isolates clustering within the large clonal clade were ST367 (278/290 isolates), which has previously been associated with cattle (Rodriguez-Rivera et al., 2014; Kovac et al., 2017). The remaining 21 isolates belonged to 7 different STs, including ST541 (3/311 isolates) and ST1593 (6/311 isolates). Mapping isolation sources onto the phylogenetic tree demonstrated that most cattle isolates clustered within the large ST367 clonal clade. The more diverse lineages included ST541 and ST1593 isolates from primarily human clinical and environmental sources (Supplementary File 1; Figure 2); 14 and 7 of the 21 isolates in this group had a source listed as ‘human clinical’ and ‘environmental’, respectively. We found that cattle isolates were significantly overrepresented within the large clonal clade, while human clinical isolates were significantly overrepresented within the more diverse lineages (false discovery rate [FDR] adjusted *p*-value < 0.05), suggesting that different genetic subtypes within this *S.* Cerro lineage are associated with humans and cattle.

### Isolates within the section Typhi *S.* Cerro lineage have an open pangenome

**Figure 3:**
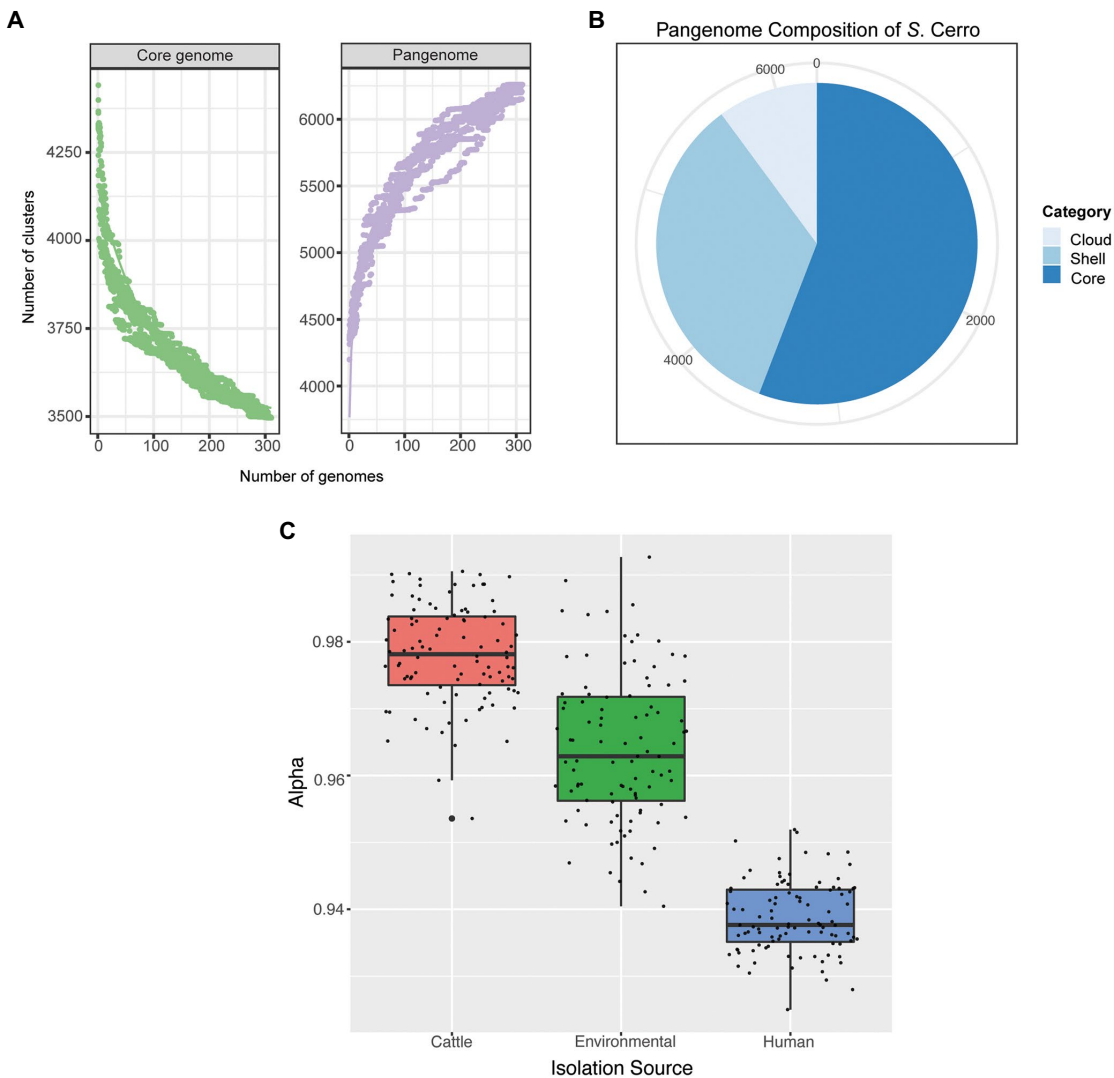
Section Typhi *S.* Cerro isolates have an open pangenome, which differs based on isolation source. **(A)** Rarefaction curves of the core and pangenome of *S.* Cerro. **(B)** Pie chart of the pangenome composition of *S.* Cerro as determined with Panaroo (Tonkin-Hill et al., 2020). Core genes are those detected in 100% of isolates included in this study, shell genes are detected in 15–99% of isolates, and cloud genes are detected in <15% of isolates. **(C)** A boxplot of alpha values of calculated Power Law Models from permutations of 15 isolates per isolation source.

As previous analyses of pangenomes in species adapted to specific niches have shown that these species often have reduced and closed pangenomes (Deng et al., 2010; McCutcheon and Moran, 2012; O’Callaghan et al., 2015), we next inferred the pangenome of section Typhi *S.* Cerro isolates and analyzed its openness to determine if pangenome size and openness can help explain *S.* Cerro’s observed association with cattle. The section Typhi *S.* Cerro lineage core genome (i.e., the number of genes detected in 100% of isolates included in this study) included 3,496 genes, while the pangenome (i.e., the number of genes detected in at least one isolate included in this study) included 6,259 genes (Supplementary File 2; Figure 3A), of which 2,130 genes were categorized as shell genes (i.e., those genes detected in 15–99% of isolates included in this study), and 633 genes were categorized as cloud genes (i.e., those genes detected in <15% of isolates included in this study; Supplementary File 2; Figure 3B). Assessment of pangenome openness using the Power Law, suggested that the pangenome of *S.* Cerro is open (*α* = 0.917), and is therefore still exchanging genetic material within its environment.

Additionally, we performed a permutation analysis in which we calculated the Power Law Model for 100 independent datasets containing 15 randomly selected isolates from each isolation source (i.e., cattle, human clinical, environmental) to determine if isolation source impacted the openness of the pangenome. The pangenomes of section Typhi *S.* Cerro isolates from all three isolation sources are all open but differ significantly by level of openness (ANOVA; *p* < 0.05). We found, on average, cattle isolates contain the least open pangenome with an average *α* value of 0.978, while human clinical isolates contain the most open pangenome with an average *α* value of 0.939 (Figure 3C).

Next, we performed genome-wide association studies (GWAS) to determine if there were any genes significantly associated with a particular isolation source or sequence type. A total of 416 genes were significantly enriched in ST367 isolates compared to non-ST367 isolates, while 500 genes were significantly enriched in non-ST367 isolates compared to ST367 isolates (FDR-adjusted *p*-value < 0.05; Supplementary File 2). Among these genes, we identified several virulence factors, including fimbriae; when we compared *S.* Cerro ST367 and non-ST367 isolates, we found that the majority of *S.* Cerro ST367 isolates encode Stk fimbria, but a small cluster of *S.* Cerro isolates with STs 769, 1,078, and 1,593 (*n* = 9) encode Sta fimbria, although the significance of this is not currently known as the binding targets of these two fimbriae remain uncharacterized (Figure 2; Supplementary File 2). Additionally, we identified several prophage-related genes (e.g., putative prophage major tail sheath protein, prophage tail fiber assembly protein TfaE, prophage integrase, prophage DNA -packing protein) that were overrepresented or underrepresented in *S.* Cerro ST367 isolates compared to other *S.* Cerro STs (Supplementary File 2). Specifically, we found that regions of the prophage SE1 genome are more abundant in *S.* Cerro ST367 isolates, while regions of the prophage ST160 genome are more abundant in non-ST367 *S.* Cerro isolates (Supplementary Figure S3). While the impact of these prophages and the genes that they carry is currently unknown and further investigation is necessary to understand what, if any, role they may play in the differences between *S.* Cerro ST367 and other sequence types, this suggests that loss and/or acquisition of prophage likely contributes to the openness of the pangenome within the section Typhi *S.* Cerro lineage.

### Cattle-associated *S.* Cerro have higher numbers of hypothetically disrupted coding sequences and show clade-specific losses of intact *sopA* and *speC*

Multiple studies have shown that disruption of coding sequences is a hallmark of host adaptation in *Salmonella* (Holt et al., 2009; Nuccio et al., 2014; Langridge et al., 2015). Therefore, we sought to identify HDCs within *S.* Cerro to characterize potential pathways associated with the serovar’s apparent adaptation to cattle. On average, section Typhi *S.* Cerro isolates contained 94 HDCs, corresponding to 2.07% of the genome. Certain HDCs were present in all 311 section Typhi *S.* Cerro isolates, including *vgrG* (SPI-19 type IV secretion system tip protein), *ddrA_1* (glycerol dehydratase reactivase subunit alpha), *vapC* (tRNA(fMet)-specific endonuclease), and *ttdA* (L(+)-tartrate dehydratase subunit alpha), suggesting that mutations in these genes were most likely acquired prior to the divergence of isolates within the section Typhi *S.* Cerro lineage (See Supplementary File 3).

To determine if the number of HDCs present within the section Typhi *S.* Cerro lineage isolates differed depending on the isolation source (i.e., cattle, environmental/food, and human clinical), we compared the HDCs among isolates within this lineage. The number of HDCs per genome differed significantly (Fisher’s exact tests, *p*-value < 0.05), with isolates from cattle containing the highest number of HDCs per genome (95 HDCs) and the highest percentage of the genome comprised of HDCs (2.10%, Table 1). Several HDCs were overrepresented in both cattle and environmental isolates compared to human clinical isolates, including the virulence factor *sopA* (SPI-1 type III secretion system effector HECT-type E3 ubiquitin transferase), and several metabolic factors including *speC* (ornithine decarboxylase) and *cfa* (cyclopropane fatty acyl phospholipid synthase; Supplementary File 3).

**Table 1:** Number of hypothetically disrupted coding sequences present in *S.* Cerro genomes.

**Isolation Source**	**Average number of genes per genome**	**Average number of HDCs per genome** ^**a**^	**Median number of HDCs per genome**	**Percent of CDS that are HDCs**	**Number of isolates per source**
Human clinical	4,424	86	86	1.94%	26
Cattle	4,523	95	95	2.10%	208
Environmental/food	4,500	92	93	2.04%	77

a Hypothetically disrupted coding sequences were determined through annotation with NCBI RAPT v. 2020-03-11, parsing resulting Genbank files for “/pseudo” identifiers, and identifying premature stop codon with BLAST and/or multiple sequence alignments.

**Figure 4:**
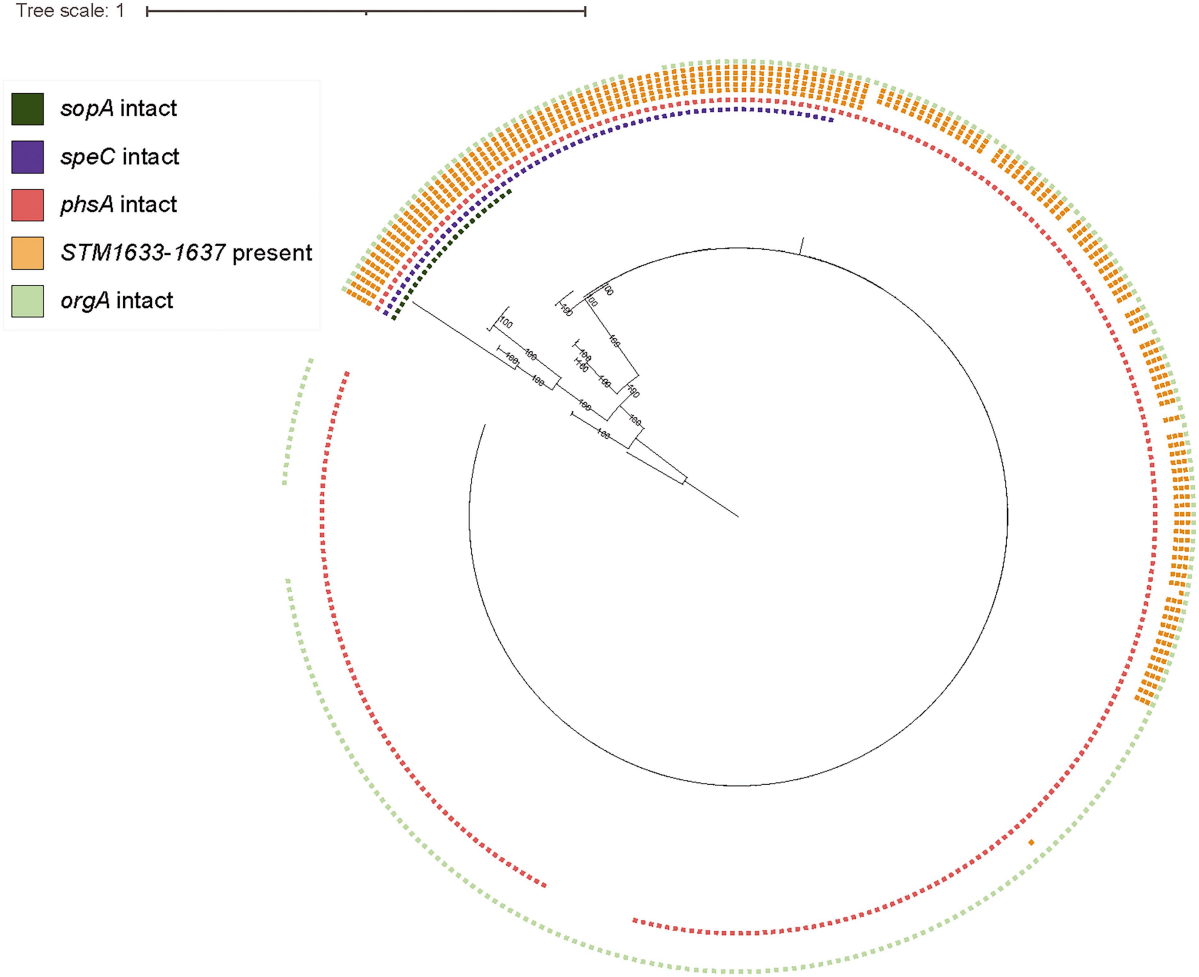
*S.* Cerro displays clade-specific PMSCs in *sopA* and *speC,* while loss of intact *phsA, orgA*, and *STM1633-1637* coincides with emergence of subclades within the ST367 clade. Maximum likelihood phylogenetic tree constructed from core SNPs for representative isolates of section Typhi *S.* Cerro SNP clusters and a previously published set of *S.* Cerro isolates (Kovac et al., 2017); *S.* Orion GCA_003874655 was used as an outgroup to root the phylogeny as it shares a most recent common ancestor with section Typhi *S.* Cerro. Squares shown to the right of the phylogeny represent the presence of intact *sopA* (dark green), *speC* (purple), *phsA* (red), *STM1633-1637* (orange; each square represents one gene), and *orgA* (light green), which are specifically shown because this and previous work (Rodriguez-Rivera et al., 2014; Kovac et al., 2017) have identified these loci as being associated with adaptation of *S.* Cerro to cattle.

Mapping of specific HDCs onto the phylogeny revealed that several HDCs were clade-specific and were present within all genomes in the clonal clade containing primarily ST367 isolates, including *sopA* and *speC* (Figure 4). Rodriguez-Rivera et al. and Kovac et al. previously identified a nonsynonymous mutation resulting in a PMSC in *sopA* at the 434th amino acid in all the isolates included in their studies (Rodriguez-Rivera et al., 2014; Kovac et al., 2017). Our HDC analyses using a larger collection of *S.* Cerro isolates confirmed that this nonsynonymous mutation follows a clade-specific pattern (Figure 4), suggesting that its acquisition accompanied the divergence of the ST367 clade. Additionally, within *speC*, we identified a nonsynonymous mutation (A to T transition at nucleotide position 1,720) resulting in a PMSC at the 573rd amino acid in 246/311 *S.* Cerro isolates. As the PMSC had not been previously established, and loss of activity of SpeC has been previously associated with host adaptation in *S.* Gallinarum (Schroll et al., 2014), we performed additional phenotypic experiments to characterize whether *S.* Cerro maintains ornithine decarboxylase activity. Both *S.* Cerro isolates containing a PMSC within *speC* and isolates encoding intact *speC* were able to metabolize ornithine into putrescine, suggesting that isolates in this clade retain ornithine decarboxylase activity, likely through the acid inducible ornithine decarboxylase, SpeF.

Finally, we determined the distribution of previously observed HDCs and gene loss in a large set of *S*. Cerro isolates (Rodriguez-Rivera et al., 2014; Kovac et al., 2017). More specifically, loss of intact D-alanine transport genes (*STM1633-1637*) occurred gradually among isolates within this clade: 21.2% of *S.* Cerro isolates maintained all 5 transport genes, while 26.1% of isolates maintained 3 D-alanine transport genes (*STM1635-1637*) and 52.7% of isolates had lost all D-alanine transport genes (Figure 4). Loss of intact *orgA,* a structural component of the SPI-1 type 3 secretion system (Lou et al., 2019), was more clade-specific, as just 10 isolates encoded a PMSC within *orgA*, and this gene was not detected in an additional 4 isolates that did not share a MRCA (Figure 4). Finally, the PMSC in *phsA* described previously (Kovac et al., 2017), was restricted to a sub-clade of 11 isolates within the ST367 lineage. Together, these results suggest that while cattle associated section Typhi *S.* Cerro isolates show degradation of genes in several metabolic pathways, consistent with host adaptation, these tend to be isolated events involving sub-clades within this lineage.

### Bayesian phylogenetic reconstruction suggests the presence of two major clades within the section Typhi *S.* Cerro ST367 lineage, one of which is associated with cattle and diverged from a MRCA that existed circa 1975

To better understand the clonal expansion of the section Typhi *S.* Cerro ST367 lineage given its association with cattle, we performed a Bayesian phylogenetic reconstruction to obtain a tip-dated phylogeny for a subset of 100 isolates (Table 2; see Materials and Methods for details about the sampling approach). The section Typhi *S.* Cerro ST367 lineage isolates were estimated to have evolved at a rate of 3.70 × 10^−7^ substitutions/site/year. Phylogenetic analyses revealed the presence of two major *S.* Cerro ST367 clades (referred to here as clades I and II) that are estimated to have shared a common ancestor circa 1920 (95% HPD: 1822–1968; Figure 5); one isolate represented a more divergent sub-lineage and was not assigned to a clade. Interestingly, clade I was composed of predominantly cattle isolates (51 of 57 total isolates; 89.5%) with a MRCA that existed circa 1975 (95% HPD: 1933–1996), while clade II contained predominantly environmental and food isolates (21 of 42 total isolates; 50%) with a MRCA that existed circa 1951 (95% HPD: 1915–1975; Figure 5; Supplementary File 4). All ST367 isolates included here contained a PMSC in *sopA,* while a PMSC in *speC* was only detected in ST367 clade I isolates (Supplementary File 4). This suggests that the PMSC in *sopA* coincided with the expansion of ST367, while the loss of *speC* coincided with the clonal expansion of *S.* Cerro ST367 clade I in cattle from a MRCA that existed circa 1975.

**Table 2:** BEAST run statistics of five combined runs using the Model 23 substitution model, relaxed lognormal molecular clock, and constant population size models.

Tree		Posterior		Clock rate		Tree model root height		Constant population size	
Mean likelihood	ESS^a^	Mean	ESS^a^	Mean	ESS^a^	Mean	ESS^a^	Mean	ESS^a^
−6,319,498	89,277	−6,320,178	1,997	5.87E−7	2,055	149	2,251	296	1,936

a ESS, effective sample size.

**Figure 5:**
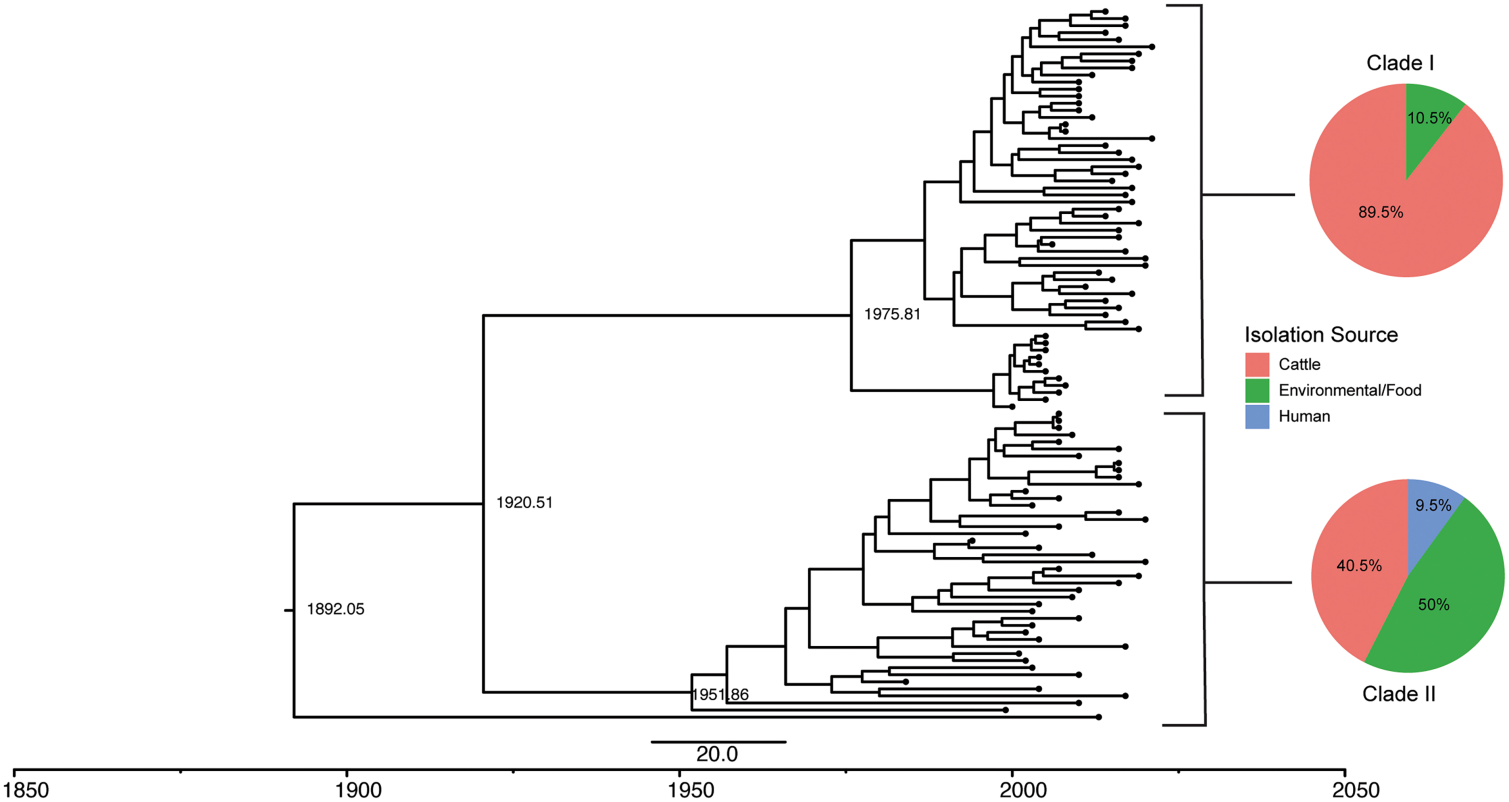
Tip-dated phylogeny of 100 *S.* Cerro ST367 isolates shows two clades, one of which is cattle associated. A rooted tree was constructed in BEAST with high quality core SNPs using the model 23 substitution model, lognormal relaxed molecular clock, and coalescent constant size population model in five independent runs of 400 million MCMC simulations. Estimated dates of divergence are displayed for the major clades. Pie charts of isolation source of all isolates in SNP clusters belonging to clades I and II are displayed next to their representative clades.

## Discussion

### While *S.* Cerro is polyphyletic, the majority of isolates sequenced represent ST367 within the section Typhi lineage

Among the 316 isolates characterized here, we demonstrate that although *S.* Cerro is polyphyletic, the majority of isolates belong to the section Typhi lineage, including the ST367 sequence type that has previously been shown to be cattle associated (Rodriguez-Rivera et al., 2014; Kovac et al., 2017). This suggests that the high prevalence of *S.* Cerro ST367 isolates represents a clonal expansion of cattle associated *S.* Cerro, while isolates representing STs 541 and 1,593 are most likely associated with isolation from humans.

The topologies of previous tip-dated phylogenies of *S.* Cerro ST367 isolates differed slightly from the tree presented in this study. The phylogenies inferred previously included smaller collections of *S.* Cerro isolates from cattle, which showed phylogeographic distributions, suggesting expansion of these clades within their respective geographic locations (Rodriguez-Rivera et al., 2014; Kovac et al., 2017). The analyses presented here included a broader diversity of *S.* Cerro isolates (including cattle, human clinical, and environmental isolates), and as such, the observed differences in topologies may reflect the number and diversity of isolates included in each study.

Importantly, the phylogenetic analyses of *S.* Cerro presented here can help inform the development of risk-based food safety regulations. Current food safety regulations in the United States, which do not acknowledge virulence differences among serovars and subtypes of *Salmonella*, have largely failed to reduce the incidence of human clinical salmonellosis (Tack et al., 2019; U.S. Department of Health and Human Services, 2020; Cohn et al., 2021a). The phylogenetic analyses presented in this study further emphasize the need to focus current regulations on subtypes and serovars of concern. In this framework, the finding that section Typhi *S.* Cerro ST367 isolates are significantly associated with isolation from cattle but not humans would deprioritize control of *S.* Cerro ST367 in beef products due to its low relative risk of causing human clinical disease. Additionally, further characterization of STs associated with humans, like STs 541 and 1,593, may allow for improved targeting of putatively human-associated subtypes of *S.* Cerro. As such, more resources could be allocated to control other subtypes and serovars common in beef products, like serovars Montevideo and Dublin, as these serovars are more likely to cause human illness (USDA, 2016; cdc.gov, 2018a).

### Section Typhi *S.* Cerro has an open pangenome, suggesting that isolates within this lineage are still exchanging genetic material, potentially mediated by the acquisition and loss of prophage

We found that *S.* Cerro has an open pangenome, which suggests this serovar is still exchanging genetic material with its environment. Pangenome openness can provide potential insights into the lifestyle of a species, serovar., or subtype. Many bacterial species, including pathogens, with closed pangenomes are typically restricted to a lifestyle in a particular host (e.g., *Buchnera aphidicola* is a symbiont of aphids), (Snipen et al., 2009; Deng et al., 2010). However, most pathogens (e.g., pathogenic *E. coli*, *Bacillus cereus*) that survive in diverse environments maintain open pangenomes (Tettelin et al., 2005; Rasko et al., 2008; Bazinet, 2017), with the openness of a pangenome being affected by the number and diversity of isolates included (Park et al., 2019). The observation that section Typhi *S.* Cerro isolates from cattle have the least open pangenome suggests that horizontal gene transfer may be occurring between *S.* Cerro and either other bacteria or mobile elements, such as bacteriophage within either the cattle gastrointestinal tract or the production environment (Mao et al., 2015). This hypothesis is further supported by the observed differences in prophage content among the diverse set of isolates characterized here, suggesting that gain and loss of prophage, such as SE1 and ST160 (or SE1 and ST160-like prophage), may explain why *S.* Cerro isolates maintain an open pangenome.

### *S.* Cerro displays a clade-specific loss of virulence and metabolic pathway genes, indicative of an adaptation to cattle

*Salmonella enterica* has evolved multiple mechanisms to successfully colonize and proliferate in a variety of hosts. Previous studies have shown that host-adapted *Salmonella* serovars that cause extraintestinal disease in humans (e.g., Typhi, Choleraesuis, Dublin) contain a number of HDCs that represent genes associated with metabolic pathways (Holt et al., 2009; Nuccio et al., 2014; Langridge et al., 2015). Among section Typhi *S.* Cerro isolates, each genome contained an average of 94 HDCs, a value that falls between that identified for host-adapted and broad host range serovars previously (Nuccio et al., 2014; Langridge et al., 2015), suggesting that isolates within the section Typhi *S.* Cerro lineage, or specific sub-clades within this lineage display some hallmarks of host-adaptation. For example, we found that the presence of the PMSC in *sopA* (Rodriguez-Rivera et al., 2014; Kovac et al., 2017) coincided with the clonal expansion of *S.* Cerro ST367, while the PMSC in *speC* occurred after the emergence of this clade.

SopA is an effector encoded within SPI-1 that facilitates colonization of the gut epithelium by mimicking two mammalian HECT E3 ubiquitin ligases, thereby stimulating the innate immune response and modulating interferon-β signaling (Zhang et al., 2006; Kamanova et al., 2016). Loss of functional SopA has been shown to decrease *Salmonella*’s ability to escape the *Salmonella* containing vacuole during infection, reduce polymorphonuclear leukocyte migration (Zhang et al., 2006), and dampen the enteropathogenic response in a bovine ligated ileal loop model (Wood et al., 2000). Loss of functional SopA in the *S.* Cerro ST367 lineage may suggest a loss in the ability to elicit gastroenteritis in humans as Typhimurium, Heidelberg, Newport, and Enteritidis maintain intact *sopA*, while serovars associated with extraintestinal disease (e.g., Dublin, Paratyphi A and Typhi) do not (Nuccio et al., 2014). Previously, we showed that ST367 *S.* Cerro has significantly reduced transcript abundances of SPI-1 genes, including *sopA*, compared to *S.* Typhimurium and *S.* Javiana (Cohn et al., 2021b), which supports the observation that *S.* Cerro ST367 isolates have a reduced ability to invade Caco-2 cells (Rodriguez-Rivera et al., 2014). Indeed, Raffatellu and colleagues demonstrated that *sopA* contributes to invasion of human colon epithelial T84 cells *in vitro* (Raffatellu et al., 2005). The observed expansion of the ST367 lineage in cattle, suggests that *sopA* is dispensable for infection of cattle, analogous to *S.* Gallinarum in chickens, which also encodes a PMSC within *sopA* (Thomson et al., 2008).

Our investigation of a host-associated, non-extraintestinal disease serovar complements previous studies examining host-adapted, extraintestinal serovars by further identifying metabolic pathways that may be differentially associated with proliferation in different hosts. Ornithine decarboxylases catalyze the synthesis of the essential polyamine putrescine from L-ornithine (Igarashi and Kashiwagi, 2010). *Salmonella enterica* encodes two ornithine decarboxylases: a constitutively expressed ornithine decarboxylase (*speC*) and an acid-inducible one (*speF*; Viala et al., 2011; Jelsbak et al., 2012). Although ornithine is present in the cattle small intestine (Sultana et al., 2003), the pH of the cattle small intestine is between 7 and 8 (Msstate.edu, 2011), suggesting that *S.* Cerro likely does not require ornithine to proliferate in the cattle intestine as *speC* is a HDC among isolates in the ST367 clonal clade that is associated with cattle; however, the activity of SpeF at this pH is unknown. Interestingly, host adapted extraintestinal serovars Typhi and Gallinarum also encode HDCs in *speC*, suggesting that SpeC activity is not essential for survival in the human or chicken gastrointestinal tract (Nuccio et al., 2014). Indeed, inactivation of *speC* in *S.* Typhimurium did not impact bacterial load in the intestine of C57/BL6 mice (Viala et al., 2011). Furthermore, gradual loss of D-alanine transport genes (*STM1634-1637*) in *S.* Cerro supports that these genes are also dispensable for *S.* Cerro proliferation in cattle. Previously, Nuccio and Baumler and Langridge et al. identified a PMSC in *pocR,* the transcriptional regulator of vitamin B12 biosynthesis (Chen et al., 1994), among extraintestinal serovars Typhi, Paratyphi A and C, and Gallinarum (Nuccio et al., 2014; Langridge et al., 2015). Vitamin B12 is important for *Salmonella* survival in the gastrointestinal tract as it is utilized as a cofactor in the metabolism of ethanolamine (Price-Carter et al., 2001). In our study, all *S.* Cerro lineages maintained vitamin B12 biosynthesis and 1,2-propanediol utilization (Supplementary File 3) pathway genes, suggesting that these metabolic pathways may facilitate survival and/or proliferation in the cattle gut.

Overall, our study provides new insights on the evolution of *S.* Cerro lineages and their apparent adaptation to cattle and potential virulence attenuation in humans. These results support that a gradual loss of select metabolic and virulence-associated functions, rather than a sudden loss of virulence, accompanied *S.* Cerro’s clonal expansion within cattle. Importantly, the insights provided here for *S.* Cerro can potentially be used as a model in studying other host adapted NTS serovars that do not cause extraintestinal disease.

## Data availability statement

The datasets presented in this study can be found in online repositories. The names of the repository/repositories and accession number(s) can be found in the article/Supplementary material.

## Author contributions

AC, RC, and MW designed the study and co-wrote, edited, and reviewed the first draft of the manuscript. AC performed the experimental, computational, and data analyses. RO, LC, and JL helped with computational analyses data analysis and edited and reviewed the final draft of the manuscript. All authors contributed to the article and approved the submitted version.

## Funding

Funding was made possible in part by grant NU50CK000528 the Integrated Food Safety Centers of Excellence. Views expressed in written materials or publications, and by speakers and moderators do not necessarily reflect the official policies of the Integrated Food Safety Centers of Excellence nor does any mention of trade names, commercial practices, or organization imply endorsement. This work is also supported by Food A Safety and Defense (A1332) grant number 2021-67017-33830/project accession no. 1024163 from the USDA National Institute of Food and Agriculture.

## Conflict of interest

The authors declare that the research was conducted in the absence of any commercial or financial relationships that could be construed as a potential conflict of interest.

## Publisher’s note

All claims expressed in this article are solely those of the authors and do not necessarily represent those of their affiliated organizations, or those of the publisher, the editors and the reviewers. Any product that may be evaluated in this article, or claim that may be made by its manufacturer, is not guaranteed or endorsed by the publisher.

## Footnotes

^1^ https://ncbi.nlm.nih.gov/pathogens

^2^ https://github.com/tseemann/mlst

^3^ https://github.com/tseemann/snippy

^4^ https://rocaplab.ocean.washington.edu/tools/genbank_to_fasta/

^5^ https://github.com/alexarcohn/salmonellacerro

## Supplementary material

SUPPLEMENTARY FIGURE S1 Phylogenetic tree of *S.* Cerro ST367 isolates. Preliminary phylogenetic tree of 100 *S.* Cerro ST367 isolates based on high quality SNPs detected using Snippy, rooted at the midpoint.

SUPPLEMENTARY FIGURE S2 The temporal signal of isolates included in BEAST analysis. The date randomization test (Duchêne et al., 2015) was utilized to test the temporal signal of isolates included in tip-dated phylogenetic analyses done with BEAST (Bouckaert et al., 2019). The rate.mean (real values and log_10_ transformed values) of the real dataset and 10 datasets with randomized dates are displayed.

SUPPLEMENTARY FIGURE S3 Length of prophages detected in section Typhi *S.* Cerro isolates differs based on sequence type. Boxplots show the average lengths (in basepairs) of genomic regions matching prophages SE1 and ST160 among 311 section Typhi *S.* Cerro isolates.
