# Supplementary figures and images for: Salmonella enterica serovar Cerro displays a phylogenetic structure and genomic features consistent with virulence attenuation and adaptation to cattle
Source: Front Microbiol. 2022 Nov 30;13:1005215. doi: 10.3389/fmicb.2022.1005215 (PMC9748477; doi:10.3389/fmicb.2022.1005215)

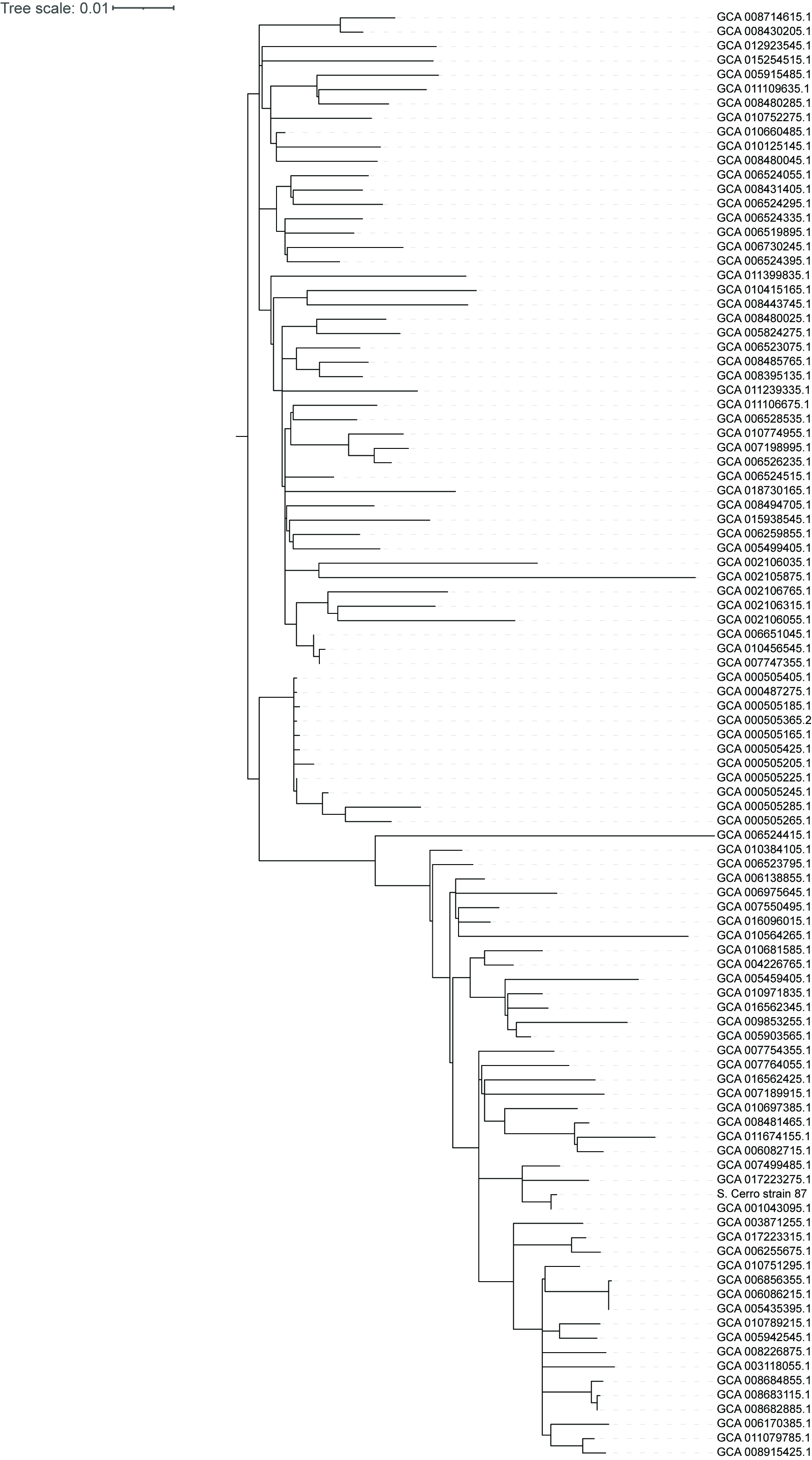

Supplement: SUPPLEMENTARY FIGURE S1 — Phylogenetic tree of S. Cerro ST367 isolates. Preliminary phylogenetic tree of 100 S. Cerro ST367 isolates based on high quality SNPs detected using Snippy, rooted at the midpoint. [file Image_1.TIF]

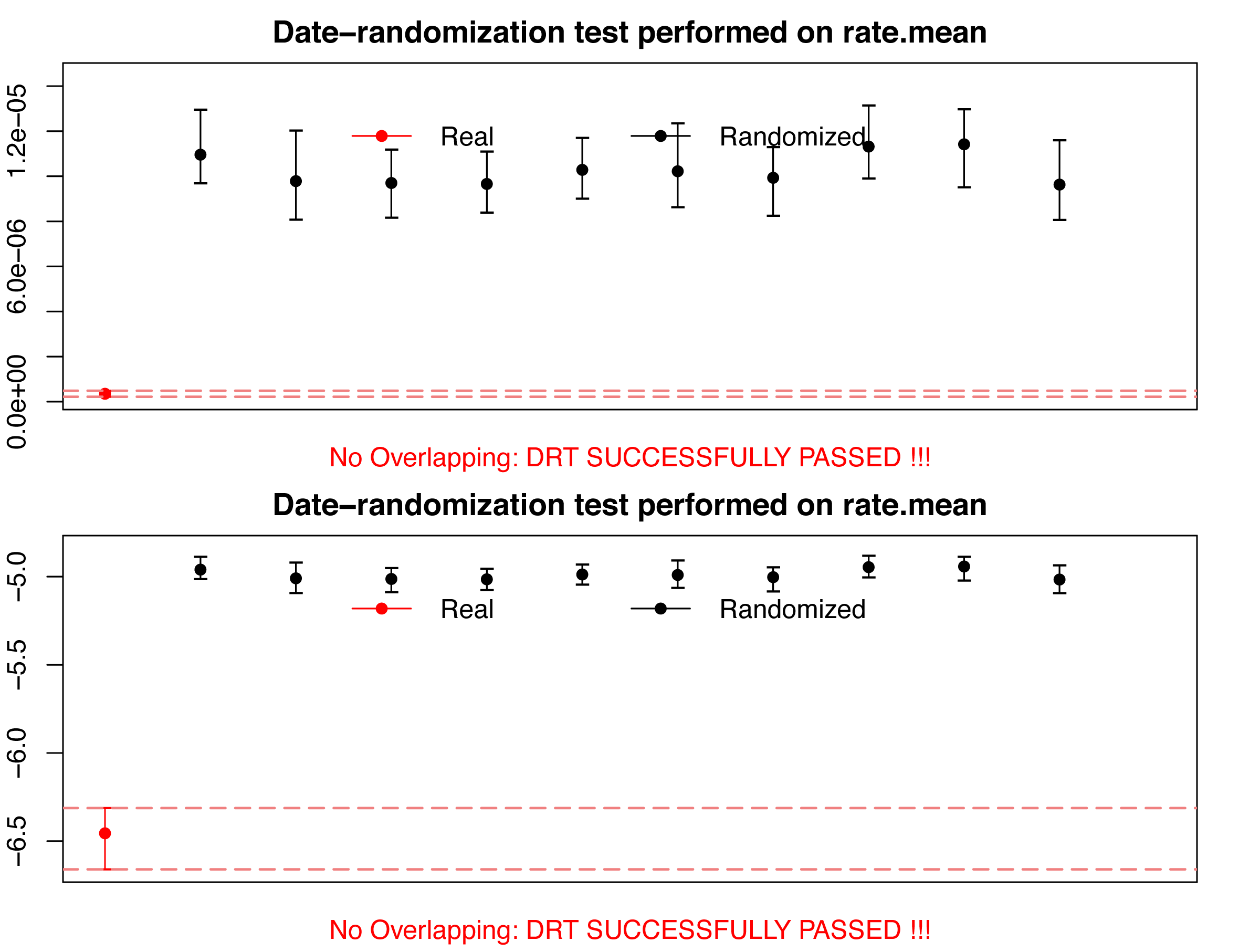

Supplement: SUPPLEMENTARY FIGURE S2 — The temporal signal of isolates included in BEAST analysis. The date randomization test (Duchêne et al., 2015) was utilized to test the temporal signal of isolates included in tip-dated phylogenetic analyses done with BEAST (Bouckaert et al., 2019). The rate.mean (real values and log10 transformed values) of the real dataset and 10 datasets with randomized dates are displayed. [file Image_2.TIF]

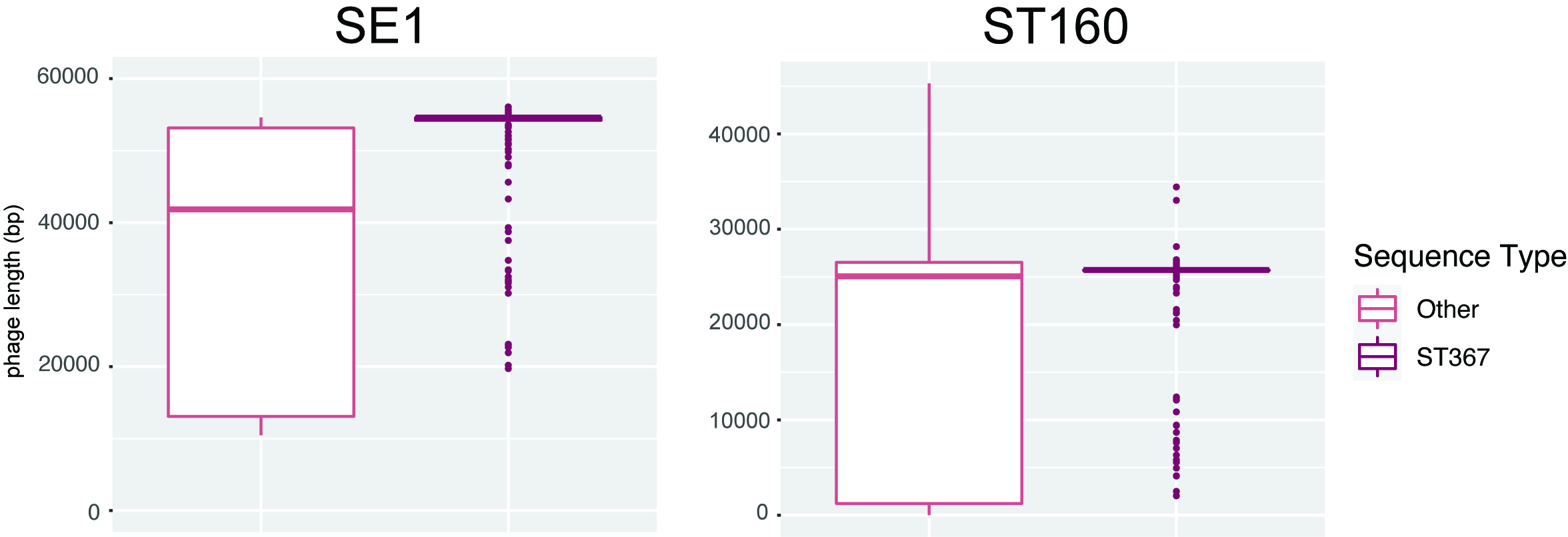

Supplement: SUPPLEMENTARY FIGURE S3 — Length of prophages detected in section Typhi S. Cerro isolates differs based on sequence type. Boxplots show the average lengths (in basepairs) of genomic regions matching prophages SE1 and ST160 among 311 section Typhi S. Cerro isolates. [file Image_3.TIF]
